# Revitalize splenic functions. Following a splenectomy for trauma, a small amount of splenic autotransplantation was performed

**DOI:** 10.1186/s12893-023-02126-z

**Published:** 2023-08-11

**Authors:** Hassan A. Saad, Rasha S Elsayed, Mohamed Riad, Ahmed K. El-Taher, Mohamed E Eraky, Ashraf Abdelmonem

**Affiliations:** Surgical Department, Faculty of Medicine, Zagazig University, Zagazig, Egypt

**Keywords:** Autotransplantation, Splenectomy, Trauma, Splenic functions

## Abstract

**Introduction:**

The spleen is a responsible significant part of the immune system; after Splenectomy following trauma, the immune system changes; splenic autotransplantation can preserve the immune system after trauma and Splenectomy.

**Background:**

Patients can be protected from immune dysfunction by autotransplanting splenic tissues after splenectomy following trauma because their immune systems and spleens are changed. Patients can gain their immune function after splenic autotransplantation.

**Methods:**

Patient classification methods are into three categories, Group A, 6 cases with auto-translation; Group B, 6 cases without transplantation; Group C, seven regular people serving as the control.

**Aim of work:**

The aim of the work is not to compare outcome methods or compare types of autotransplantation; This work aims to document postoperative radiological, immunological, clinical, and hematological investigations. We concentrated on the results of investigations more than the types of operation or approach or types of autotransplantation.

**Results:**

We showed that, after comparing each group with normal individuals subjects, patients who did not undergo autotransplantation had significantly higher platelet counts, a more significant percentage of micronucleated reticulocytes, increased levels of naive B lymphocytes, changes in class-switched memory and class-unswitched memory B cells, and higher levels of PD1 on CD8 + T lymphocytes. Nevertheless, neither splenic autotransplant patients nor the average general population showed any appreciable variations in any of the parameters.

**Conclusions:**

Spleen’s activities with adequate hemocatheter activity and recovery of the immunological deficit after splenic autotransplantation.

## Introduction

About 25% of all solid abdominal organs have splenic damage following blunt abdominal injuries. The thin capsule and fragile structure of the spleen make marsilpiazation difficult [1]. However, after the procedure and splenic removal, the immunization became impaired, making the patients more susceptible to post-splenectomy infection [2]. Because of the fatality rate of 7–18%, splenic transplantation is the best option for preventing immune changes[3]. In the past (1946), splenic slices were implanted in the omentum [4]. Still, this procedure had several postoperative complications such as infection, intestinal obstruction, and bleeding minor trauma that disappeared because of other technical methods advancements[5]. In 1977, it was reported that non-operational management (NOM) was the best choice for preserving immunological functions [6]. We just published a new method for preserving the health of a single transplanted piece of splenic tissue. This transplant was done in the spleen’s natural position, avoiding all the problems with earlier procedures that had to do with the quantity and positioning of the slices that were being transplanted. According to the preliminary report, this technique is problem-free [7].

Although there are apparent advantages of NOM (with or without AE) in terms of avoiding splenectomy and complications associated with a laparotomy, there are numerous possible disadvantages to NOM. These include its costs and morbidities, the fact that it is not always technically feasible or successful, the need for strict patient behaviour and close expert monitoring, the fact that it does not completely prevent delayed splenic rupture or haemorrhage, and the constant availability of an operating room and operative team [4].

Blunt or penetrating splenic injury requiring surgical exploration for diaphragmatic or hollow-viscus injuries, high-grade blunt splenic injury with unavailable, contraindicated, unfeasible, or unsuccessful NOM and AE, and all complications following AE such as pseudoaneurysms, splenic infarction or abscess, and delayed rupture are all indications for splenectomy in hemodynamically normal patients. NOM is not recommended for patients who are unable or unable to adhere to the rigorous NOM conduct and activity limits (e.g., mentally challenged, homeless, self-employed, professional sports), as well as those who have an inaccurate examination, generally due to concomitant injuries and intubation [5].

## Materials and methods

An analysis of the materials and techniques was done Between 2020 and 2023; the Department of Surgery at Zagzig University studied 6 cases (group A) that underwent splenectomy autotransplantation. This study was contrasted with (group B), which involved six patients who underwent splenectomy without autotransplantation, and (group C), which included seven physically fit individuals.

### General and surgical data

After patients gave their permission, data on age, sex, complications, indications for splenectomy, and degree of splenic trauma according to the Association of Surgeons Trauma (ASST) [8], blood loss, operation time, length of hospital stay, postoperative complications, lab work, and platelet counts were calculated for both groups. All patients approved and accepted the use of their medical records for research, according to the 1969 Declaration of Helsinki.

Personal integrity and physical health for group A.

**Fig. 1:**
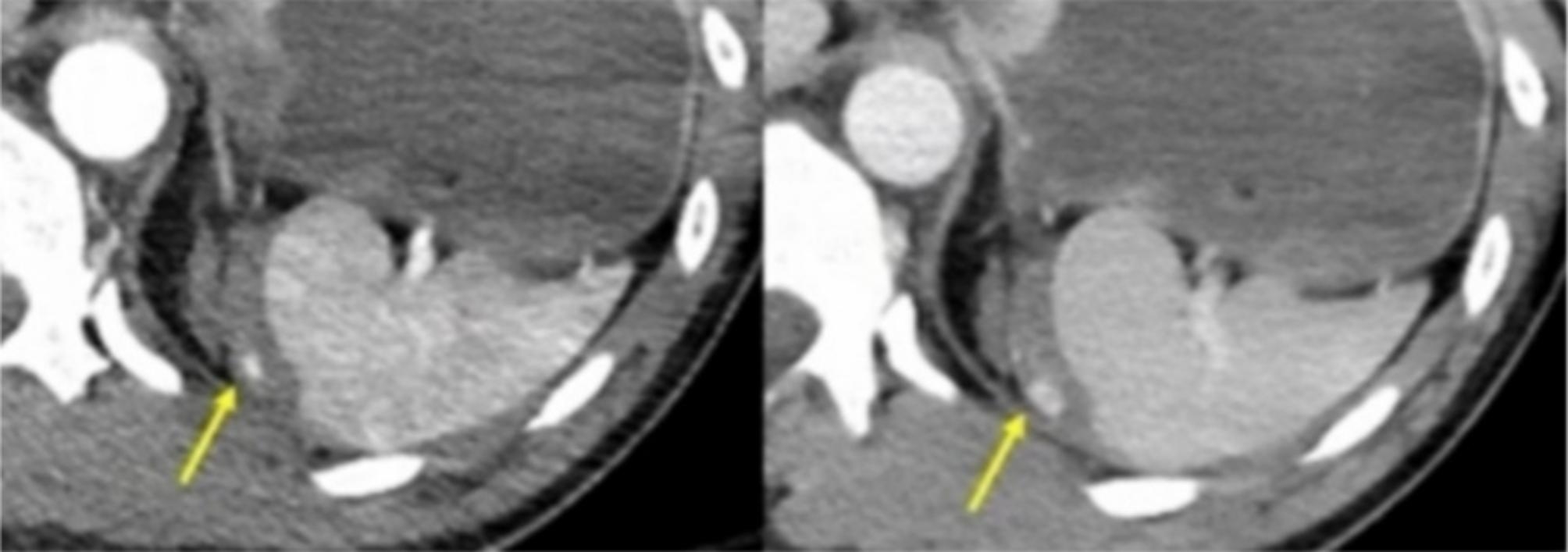
CT for partial splenic tear

Studied morphology by (CT) during follow-up and postoperative see Fig. 1, showing the degree of splenic tear. And Fig. 1. Showing partial splenic tear after acute car accident trauma.

### Procedures and techniques

**Fig. 2:**
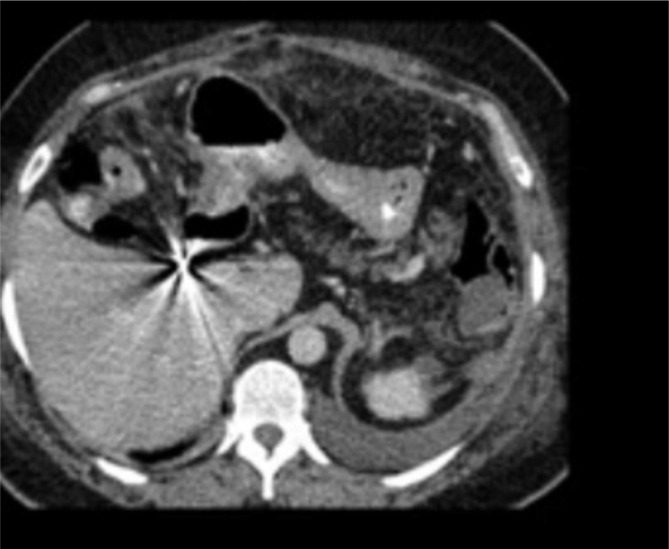
CT contrast post splenectomy after truma

The procedure involves inserting a single segment of splenic tissue, weighing 35 gm and measuring 4 × 3 × 2 centimeters, inside the more significant pedunculated edge of the omentum from the pouch. The omental pouch was attached with special Prolene stitches in the surrounding region, fixed beneath the left couplet of the diaphragm, using Prolene knotted 4/0 (without traction) to attain its anatomical site. Figure 2 showings CT contrast postsplenectomy.

### Radiological information

**Fig. 3:**
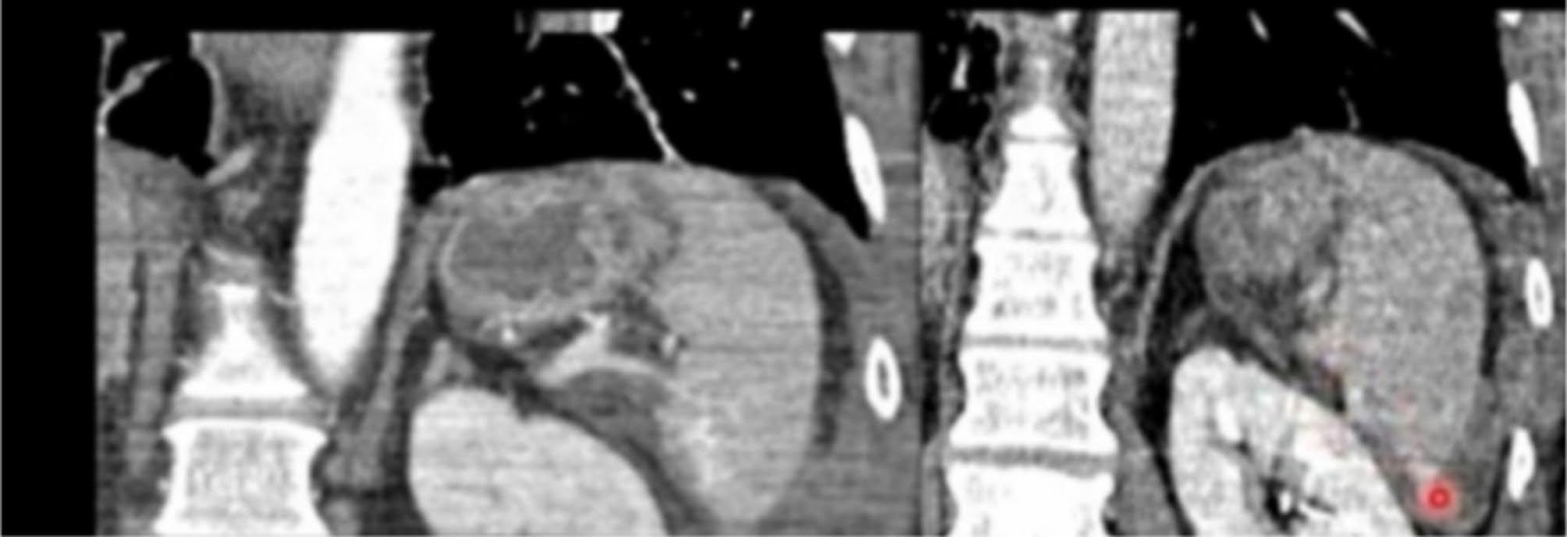
CT contrast showing grade 5 tear after falling from up

The primary goal of CT for group A following surgery was to evaluate the size and location of the splenic implanted tissues and to show afferent and efferent blood flow through contrast and angiogenesis during the basal, arterial, and portal phases. With the “smart-prep” and regard to the celiac tripod’s level, an arterial gaining was carried out. At the time of acquiring, or acquisition, the contrast flow rate was 3.5 ml/s, and the layer its thickness was 1.25 mm. The quantity of the nonionic contrast was 350/370 mg/ml. According to age and weight, a suitable degree of contrast was chosen. The portal phase took place 70 s after the infusion started, and multiplane elaborations of the arterial and portal phases were carried out. Figure 3. Grad 5 splenic trauma after falling from 4 floor.

### Hematological and immunological data

To assess immune function, hematological and immunological data flow cytometry is used to quantify the B and T lymphocyte parameter from blood samples: reticulocytes (MN-RET) count and hemocatheterisis-related infection.

The blood sample was collected in EDTA containers and was ready in 2 h. All samples were examined by a NAVIOS F.cytometer (Beckman colter), and for each examination, 100,000 occurrences were done.

### Micronucleated reticulocytes (MN-RET)

Dertinger SD is used to quantify or assess micronucleated reticulocytes. For each analysis [9], The samples were blended with anti-CD71 FITC and anti-CD42b-PE (monoclonal antibodies) (both from Beckman Coulter), then bicarbonate-buffered used to be as soon as used to wash it with saline solution, accompanied through the use of RNase treatment. Ultracold methanol (IT. Baker) is constantly combined with heparin and saved at 75 °C (Sigma). The remedy blanketed the monoclonal anti-CD42b. to now no longer perform platelets. After 20 min barring light, we add 1 ml of ice-cold propidium iodide reply (1.25 mg PI (Sigma)/ml bicarbonate-buffered saline). MN-RETs confirmed RBCs that had been top-notch for CD71, on the other hand, bad for CD42b.

Triple examination and staining of the sample. The erythrocyte fraction was MN-RET, negative for CD42b but positive for CD71 and propidium iodide.

### Studied B cells

CD19 (ECD), IgD (FITC), CD21 (PE), CD38 (APC-750), CD27 (PC7), CD24 (APC), (Pacific Blue), IgM, and CD45 had been the eight monoclonal antibodies used to characterize B cells (Krome Orange).

B-naive (CD19 CD27IgD ) and B-class-unswitched memory (CD19 CD27 CD38IgM IgD ) had been the B-subpopulations that ought to be differentiated from each brilliant with the aid of using way of IgM, IgD, CD38, and CD27. After gating on the CD19 positive, the fantastic expression of B class-switched reminiscence (CD19 CD27 CD38IgMIgD) emerged.

### Studies of T cells

The Dura Clone IM T Cells bundle can be used to pick out the T cell subgroup. We had been in a position to distinguish lymphocytes of CD4 and CD8 T lymphocytes thru the gate on CD3 positive; consequently, the expression of PD1 on these two mobile firms used to be assessed. CD45RA and CCR7. According to the manufacturer’s recommendations, we use monoclonal antibodies in opposition to the following CD4 T lymphocytes: CD45RA (FITC), CCR7 (CD197) (PE), CD28 (ECD), PD1 (PC5.5), CD27 (PC7), CD4 (APC), CD8 (A700), CD3 (APC-750), CD57 (Pacific Blue), and CD45 (Krome Orange). The CD4 CCR7 CD45RA naïve T cells, CD4 CCR7 CD45RA- central T cells, and CD4 CCR7 CD45RA- reminiscence T cells.

### Statistical files

from all cases [19], qualitative data, percentages, and frequencies had been all decided for all alterations.

To look at firms o,f facts with qualitative and quantitative data, respectively, The **Kruskal-Wallis** seems to be at used to be as soon as used to specify the rate as the mean or median value. (25–75%). Be considerable for p values 0.05.

## Results

### Clinical outcomes

There were 19 participants in the study: 6 cases in groups A and B combined and 7 in group C; however, two were not further studied in our work; one was arrested (died), and one was not followed because he was in the far outlying area.

in Every group of personal and surgical traits Table 1 shows that all patients had spleen trauma of grades IV-V according to the AAST categorization showing in(Fig. 3). No statistical differences between groups A, B, and C In terms of gender identity, age, period of hospitality, surgical problems, or measurement of bleeding.

**Table 1:** Methods of autogenous splenic transplantation

Patel et al.48 1981	1981	4	Omentum	Two sections of the whole spleen	
Velcek et al.Y0 198	1982	3	Omentum	15–20 Sect. 15 × 15×mm	
Moore et al.1	1984	3	Omentum	Five Sect. 40 × 40 × 3 mm	
During et al.5	1984	9	Omentum	Splenic homogenate in 10 cm3 pouches 1/3 total spleen	
Nielsen et al.1	1984	6	Omentum	Splenic homogenate in 10 cm3 pouches 1/3 total spleen	
Nicholson et al.5	1986	6	Omentum	2–3 mm cubes 30–50 g	
Traub et al.1	1987	7	Periperitoneal	25–30 g thin sections	
Buyukunal et al.1	1987	16	Omentum	2–4 Sect. 30 × 50 × 5 mm	
Mizrahi et al.4	1989	10	Omentum	10 Omentum 3 mm thick section 50gm	

### Hematological outcome

#### 1- Reticulocytes outcome

Patients in group B (not autotransplant) had increased median ranges of MN-RET in contrast to group C (standard control) (p = 0.002), while these in group A (autotransplanted) were no longer statisignificantlyremarkably different from group C. (Table 2).

**Table 2:** Characteristics of the three groups of patients Patient Sex Age

Patient/age	Sex.	Patient/age	Causes and indication of Splenectomy	Time of surgical intervention	Transfer	Blood loss	Postoperative complication
1 A 28	F	1 A 28	Car Accident	90 min	4bags	1000ml	Non. 570.000
2 A. 52	F	2 A. 52	Car Accident	105 min	3bags	500ml	Non
3 A 54	M	3 A 54	Car Accident	120 min	4bags	2000ml	Non
4 A 54	M	4 A 54	Accidental fall	90 min	2bags	500	Pulmonary thickening
5 A 46	M	5 A 46	Accidental fall	120 min	4bags	1000ml	Non
6 A 26	M	6 A 26	Skiing accident	95 min	Non	500ml	Non
1B 25	F	1B 25	Car Accident	140 min	Non	1000ml	Non
2B 56	F	2B 56	Car Accident	155 min	4bags	500ml	Non
3B 24	M	3B 24	Car Accident	185 min	2bags	1000ml	Non
4B 54	F	4B 54	Car Accident	70 min	Non	1000ml	Pulmonary thickening
5B 41	F	5B 41	Car Accident	75 min	Nom	500ml	Non
6B 52	F	6B 52	Car Accident	80 min	Non	500ml	Non
1 C. 30	M	1 C. 30					
2 C. 28	F	2 C. 28					
3 C. 31	F	3 C. 31					
4 C. 30	M	4 C. 30					
5 C. 29 6 C. 30 7 C. 27	F M M	5 C. 29 6 C. 30 7 C. 27					

### 2- B cell- numbers

B cells that precise CD19 have been determined in all companies without any discernible differences. However, when compared, group C and group B had higher degrees of naive B lymphocytes (p = 0.01) but reduced tiers of class-switched reminiscence (p = 0.001) and class-unswitched reminiscence (p = 0.002), but in group A and C had no differences in B cell (Table 3).

**Table 3:** Characteristics of the three groups, demographic, platelet, thrombocytes, splenic size, and year of splenectomy

Age(years) / No. Of platelets	Year of Splenectomy	Thrombothytosis	Size of splenic transplants
1 A. 28 / 570.000	2017	No	24 × 22 mm
2 A. 52 / 2389.000	2010	No	15 × 10 mm
3 A. 54/ 318,000	2010	No	25 × 25 mm
4 A. 54 /245,000	2015	No	43 × 37 mm
5 A. 46 / 379,000	2012	No	15 × 22 mm
6 A. 26 / 237,000	2015	Yes	
1B. 25/ 581.000	2017	Yes	
2B. 56–424.000	2011	Yes	
3B. 24/ 422.000	2017	Yes	
4B. 54 / 229.000	2016	Yes	
5B. 43/ 422.000	2017		
6B. 52/206.000	2016		
1 C. 30/ 299.000	2015		
2 C. 28/216.000			
3 C. 31/ 200.000			
4 C. 30 / 219.000			
5 C. 29/ 231.000			
6 C. 30/ 204.000			
7 C. 27/ 305.000			

### 3-T- cell analysis

T cellular populations.

similar in the three classes when. The same is real for CD3 T cells, CD3 CD8, and CD3 CD4 T-subsets.

There are no longer any variations in the percentage of CD4 T lymphocytes (CD4 CCR7 CD45RA), central memory T cells (CD4 CCR7 CD45RA), or effector memory T cells (CD4 CCR7CD45RA) between the groups. But it increased PD1 on T lymphocytes CD8 and CD4 in group B sufferers as opposed to group C cases (p = 0.08 and 0.05, respectively, without significant differences between groups A and C (Table 4).

**Table 4:** Statistical evaluation of blood’s peripheral cellular groups

Cells population compared	Group A	Group B	Group C	P value	
%MN	1.3 (1–1.6)	2.7 (2.1–3.4)	0.7 (0.4–1.3)	B vs. C p = 0.002 A vs. C p = 0.07	
B cell populations % B cells CD19+	10.5 (7.4–14.4)	10.3 (5.85–12.4)	8.4 (6.7–9.7)	B vs. C p = 0.4 A vs. C p = 0.1	
% B cells naive CD19 + CD27-IgD+	58 (53.5–62.6)	64.5 (58–70.9)	54.6 (46.2–59.2)	B vs. C p = 0.01 A vs. C p = 0.4	
Class-switched memory CD19 + IgD-IgM- CD38-CD27+	15 (11.1–20.6)	12.8 (10.5–14)	54.6 (46.2–59.2)	B vs. C p = 0.001 A vs. C p = 0.06	
% B cells Class-unswitched memory CD19 + IgD + IgM + CD38-CD27+	18.7 (14.5–23.3)	15.4 (11.8–18.5)	22.6 (20.1–25.8)	B vs. C p = 0.002 A vs. C p = 0.05	
Tcell population%					
T cells CD3+	62(53.5–75.1)	75.3(56.3–79.6)	76.8(60.1–82.1)	B vs. C p = 0.6 A vs. C p = 0.4	
% T cells CD4+	62.7(59.9–67.2)	61.8(53.4–67.2)	57.5(55–65)	B vs. C p = 0.6 A vs. C p = 0.4	
% T cells CD8+	30(24–31.6)	30.7(24.6–35.3)	32.1(28.6–35)	B vs. C p = 0.5 A vs. C p = 0.1	
% T cells CD4 + PD1+	20.5(13.1–29.7)	29.7(26.7–30.5)	23.5(13.1–25.6)	B vs. C p = 0.05 A vs. C p = 0.8	
% Naive-T cells CD4 + CCR7 + CD45RA+	26.9(20.6–37.4)	37.1(31.6–48.2)	27.3 (24–39.4)	B vs. C p = 0.4 A vs. C p = 0.6	
% effector memory T cells CD4 + CCR7 − CD45RA−	22.7 (14.5–29.3)	15.8(11.1–25.7)	22.2(14.9–24.7)	B vs. C p = 0.1 A vs. C p = 0.7	
% central memory T cells CD4 + CCR7 + CD45RA-	48.6 (42.5–55.8)	42(34.1–43.2)	46.5(40.1–58.2)		

Splenic tissue auto-transplanted into Group A

Splenectomy in Group B

Healthy individuals make up Group C

The median is used to convey values. (25th and 75th percentiles)

## Discussion

As WSES guidelines are still being considered, NOM, involving splenic artery embolization, has lately been broadened to all steady patients, despite AAST grade [10]. Still, this type of therapy can only be given in an intensely specialized trauma center. NOM for grade IV-V AAST is still challenging to perform in outlying hospitals worldwide, and splenectomy is frequently seen as the preferable surgery for both the patient and the surgeon [2].

OPSI is the miserable and risky complication of splenectomy, frequently resulting in death. After two years, that happened [11]. A 1999 research found that autotransplantation had a noticeable impact on patients who had undergone splenectomy immune fitness [12].

The scientific world is gradually coming around to laparoscopic splenectomy for trauma patients. Only short case series with limited scientific evidence exists due to the unpredictable nature of trauma [13]. Nonetheless, all published data and the current study show that laparoscopic splenectomy is safe and feasible in hemodynamically non-compromised patients with splenic injuries not amenable to NOM (or failed NOM) and adjuncts such as angioembolization, regardless of patient age, the severity of the trauma, or presence of associated injuries. When compared to standard open splenectomy, LS is associated with non-inferior morbidity and mortality and significantly improved post-operative recovery; however, the potential benefits and safety of minimally invasive surgery must be considered about the level of expertise of the institution, the availability of adequate laparoscopic equipment, and, most importantly, the presence of an experienced and skilled laparoscopic surgeon [8].

To properly examine this cutting-edge issue and characterize the immediate and long-term benefits of laparoscopic splenectomy for trauma, prospective or randomized controlled trials in patients with hemodynamically non-compromised or ‘quasi-stable’ splenic injuries are required [13].

Furthermore, one patient died from follow-up, not from OPSS. There are no studies that show whether splenectomy with autologous transplantation alters the immune system’s response to immunization or not. Given the small number of participants in the research and the fact that the participants’ immune changes had already been restored, vaccinations may not be required [12, 13].

Maintaining healthy immune functions in patients with spleen autotransplantation depends on spleen viability by proper fixation in the anatomical site [14]. However, improper techniques can lead to complications; by the prevention of splenic torsion or strangulation, we avoided those problems because it was successfully fixed to its anatomical location under the left couple after splenic omental pouching without experiencing the problems associated with single segment fixation so no others complication detected in our group due to single, well fixed and suitable anatomical pouched pice of the spleen.

The angiogenesis in splenic tissues is evident in the CT image. With typical normal platelet in group A, but in group B had a continuous elevation of the platelet counts due to lost spleen and emo catheter function; there were no statistical variations between the two groups due to the small number of patients.

Our research has concentrated on the immune reaction and re-emergence of implant function outside surgical technique. MN-RET, which increased in group B and confirmed results from a different review, showed that patients had lost splenic function without implants. Confirming outcome data in another lecture [15].

In contrast, group A patients had had an MN-RET as (group C), indicating that in these patients restored splenic hemocatheretic activity.

The spleen is responsible for B cell maturation; in our study, the declined group B (non-transplanted group) [16].in group B continued the increase naive B cell with decreased Class-switched and unswitched memory; in contrast, the group A patients did not present any significant differences for all of the raised B lymphocyte subsets in comparison to group A.

Due to the interaction between B cells and follicular helper T cells as an essential step in the formation of memory B cell counterparts or plasma cells that have high affinity to class-switched antibodies, the spleen maturation and synthesis of freshly formed B lymphocytes and IgD that support our research [16]. Normal subjects’ B- naive cells multiply and differentiate into plasma B cells in reaction to T-cells, which causes secondary lymphoid organs to form. A T-cell mutation that causes impairment results in a defect in the immune system [17].

In our research, we found that autotransplantation cells caused a reduction in memory B. More quickly than naïve B cells, plasma cells and memory B cells respond to pathogens, boosting immunity and defending against them. Due to reduced memory B-cells, the functional GC response is altered [18], and iso, autoimmune issues such as chronic granulomatous.

Memory B cell was highly reduced in peripheral blood in a patient with congenital asplenic [19]. These patients lose their immune systems and defense, making them more susceptible to recurrent infection [20]. Also, abnormal B- Cell found in their peripheral blood in special immune defense [21]. We expected different GC responses in certain immune disorders [22]. Individuals with particular granulomatous immune disorder CGD had low mature B cells and high naive- B cells [23]. Increased T cells in no auto-transplanted patients but not in autotransplantation, with CD8 + and CD4 + T lymphocytes carrying the PD1 marker.

Programmed cell death-1 (PD-1) is a member of the B7-CD28 subfamily of immunoreceptors that sends out unfavorable signals when it interacts with its ligand (PD-L1) [24]. It plays a part in controlling the structures of T-cell responsiveness. It is widely known that blocking the PD-1/PD-L1 axis enhances the immunological response to antigens given by dendritic cell populations and that PD-1 is a critical immune regulatory mechanism that inhibits autoimmunity [25].

However, high PD-1 expression on T cell surfaces impairs these cells’ capacity to fight off cancer and infectious diseases. According to research, PD-1 is highly expressed in T cells in septic patients [26].

More significantly, PD-L1 is conveyed on the surface of many tumor cells, a blatant mechanism by which tumors escape immune cell control. T lymphocytes in the tumor context are often PD-1 positive. They are retired lymphocytes, which indicate diminished T cell responsiveness, diminished generation of beneficial cytokines, and a lack of cytotoxic action [27]. Targeting the PD-1 immune checkpoint in treating numerous advanced malignancies refractory to traditional chemotherapy has demonstrated great clinical success [28].

## Conclusion

Splenectomized patients had poor and impaired immune function and decreased function of B and T lymphocytes. So, the splenic function of hemocatheric function after splenectomy with autotransplantation patients gained immune function and splenic hemocatheretic function close to normal individuals. But there are only a few patients number in the present research. With these early findings, a sizable multicenter investigation can now be recommended.

## Data Availability

The database, including figures and materials under our review with the corresponding author when your request. All authors shared the database and work.

## Abbreviations

ASST: American Association Surgeons Trauma
CGD: Chronic granulomatous disease
CT: Computed tomography
MN-RET: Micronucleated reticulocytes
NOM: Non-operative management
OPSI: Overwhelming post-splenectomy infection
PD-1: Programmed cell death-1
PD-L1: Programmed cell death-ligand 1
SEA: Splenic arterial embolization
